# Addressing future food demand in The Gambia: can increased crop productivity and climate change adaptation close the supply–demand gap?

**DOI:** 10.1007/s12571-024-01444-1

**Published:** 2024-04-26

**Authors:** Tony W. Carr, Felicity Addo, Amanda Palazzo, Petr Havlik, Katya Pérez-Guzmán, Zakari Ali, Rosemary Green, Genevieve Hadida, Alcade C. Segnon, Robert Zougmoré, Pauline Scheelbeek

**Affiliations:** 1 Department of Population Health, London School of Hygiene & Tropical Medicine, London, UK; 2 International Institute for Applied Systems Analysis, Laxenburg, Austria; 3 Nutrition & Planetary Health Theme, MRC Unit The Gambia at the London, School of Hygiene and Tropical Medicine, Banjul, The Gambia; 4 Centre on Climate Change and Planetary Health, London School of Hygiene & Tropical Medicine, London, UK; 5 International Center for Tropical Agriculture (CIAT), Dakar, Senegal; 6 Faculty of Agronomic Sciences, University of Abomey-Calavi, Cotonou, Benin

**Keywords:** Food security, Food system model, Climate change adaptation, Crop productivity, Diets, Food imports

## Abstract

With rising demand for food and the threats posed by climate change, The Gambia faces significant challenges in ensuring sufficient and nutritious food for its population. To address these challenges, there is a need to increase domestic food production while limiting deforestation and land degradation. In this study, we modified the FABLE Calculator, a food and land-use system model, to focus on The Gambia to simulate scenarios for future food demand and increasing domestic food production. We considered the impacts of climate change on crops, the adoption of climate change adaptation techniques, as well as the potential of enhanced fertiliser use and irrigation to boost crop productivity, and assessed whether these measures would be sufficient to meet the projected increase in food demand. Our results indicate that domestic food production on existing cropland will not be sufficient to meet national food demand by 2050, leading to a significant supply–demand gap. However, investments in fertiliser availability and the development of sustainable irrigation infrastructure, coupled with climate change adaptation strategies like the adoption of climate-resilient crop varieties and optimised planting dates, could halve this gap. Addressing the remaining gap will require additional strategies, such as increasing imports, expanding cropland, or prioritising the production of domestic food crops over export crops. Given the critical role imports play in The Gambia’s food supply, it is essential to ensure a robust flow of food imports by diversifying partners and addressing regional trade barriers. Our study highlights the urgent need for sustained investment and policy support to enhance domestic food production and food imports to secure sufficient and healthy food supplies amidst growing demand and climate change challenges.

**Supplementary Information:**

The online version contains supplementary material available at 10.1007/s12571-024-01444-1.

## Introduction

West Africa is facing significant challenges in guaranteeing food security for its rapidly growing populations. The ever-increasing demand for food is accompanied by increasing vulnerability of food production due to environmental changes, particularly climate change, which is severely affecting the region (Trisos et al., 2022). Additionally, widespread land degradation, including depleted soil fertility, has reduced yields in agriculture (Stewart et al., 2020).

The Gambia is facing particularly substantial food availability issues as its agricultural sector has lower productivity than other West African countries (World Bank, 2019). The Gambian agricultural sector is an important contributor to the country's economy and employs nearly half of the workforce (Cisse et al., 2021). Crop production accounts for the majority of agricultural GDP, with main crops including groundnuts, millet, rice, maize, and sorghum. However, apart from groundnuts grown under resource-intensive conditions for the export market, most crops are produced through traditional rain-fed agriculture and subsistence farming, where farmers are often restricted by low soil fertility and limited access to inputs (World Bank, 2019).

Agricultural production in The Gambia has seen consistent growth in recent decades. However, the increase has not been achieved from maximising productivity on existing cropland, but rather from expansion into conserved forests (World Bank, 2019). If current deforestation trends continue, The Gambia's forests could be completely depleted by 2050 (MECCNAR, 2021). This presents a challenge for the country as it strives to balance its goals of preserving its forests, waters, wildlife, wetlands, and biodiversity with the need to increase food production (MECCNAR, 2021).

The country's dependence on food imports is substantial, with only half of current food demand being met with domestic production (Cisse et al., 2021; MECCNAR, 2021). For instance, the country imports 83% of its rice, a staple food for most Gambian households (World Bank, 2019). The increasing demand for food has led to rising imports, making The Gambia vulnerable to external disruptions in food supply due to climate change or other shocks.

Undernutrition continues to be a problem in The Gambia; however, diet-related non-communicable diseases (NCDs) have also emerged due to changes in diets and lifestyles with increasing incomes (Ali et al., 2023). A recent study found that the average Gambian diet has low adherence to global recommendations for healthy and sustainable diets, with a dominance of refined grains and added sugars exceeding guidelines, and low consumption of nutritionally important food groups, such as fruits and vegetables (Ali et al., 2022). To improve the nutritional status of the population, a National Nutrition Policy has been established, aiming to improve access to sufficient and healthy food (NaNA, 2021).

To achieve The Gambia's nutrition policy objectives and maximise co-benefits for health, environmental sustainability and socio-economic development, a holistic understanding of food system interactions is key. Integrated food system models can aid in linking The Gambia's food policy objectives by providing a broad overview of the food and land-use system and its interrelated components. These models simulate the complex interactions between system components impacting food supply and demand at national or regional levels, identifying trade-offs and synergies between different policy goals (Havlík et al., 2014; Valin et al., 2014; van Soest et al., 2019). Careful scenario analysis using integrated food system models can support informed decision-making, allowing policymakers to assess policy impacts on food security, nutrition, trade, and the environment, before implementation (Palazzo et al., 2017).

In this study, we aim to gain a better understanding of the future of The Gambia's food and land-use system and its impact on food availability by adapting the FABLE Calculator, a model developed by Mosnier et al., (2020, 2023) to represent the food system and its interactions with land-use and other sustainability indicators at the national level. Utilising scenarios co-developed with Gambian stakeholders and the integrated food system modelling approach, we project future food demand due to population growth and assess the feasibility of boosting domestic food production to decrease The Gambia's reliance on imports for its food supply. This assessment considers the effects of climate change, climate change adaptation measures and improved access to fertiliser and irrigation on crop production. Our analysis provides valuable insights into the food system challenges faced by The Gambia and helps to determine priorities for ensuring a sufficient and nutritious food supply in the future. In addition, this study presents a nationally adapted integrated modelling framework that can be employed by other countries facing similar challenges in providing sufficient and healthy food to their populations.

## Methods

### The FABLE calculator

The FABLE (Food, Agriculture, Biodiversity, Land-Use, and Energy) Calculator^1^ is an open-source Excel-based accounting model developed by Mosnier et al. (2020) to analyse the potential evolution of food and land-use systems from 2000 to 2050 with the understanding that agriculture plays the most significant role in land-use change. The model approximates the agricultural demand and supply from 2015 onward in five-year time steps, using historical FAOSTAT data (from 2000–2010) to calibrate the model. By combining various scenarios, the model simulates the potential future trajectories of food demand and land-use sectors, assessing their impacts on food security, production, trade, land use, land use change, greenhouse gas emissions, biodiversity, and water footprint.

To assess future trajectories, the FABLE Calculator requires users to modify the drivers of system change, which are influenced by the choice and combination of model parameters and assumptions (i.e., scenarios). In this context, a user-defined combination of scenarios, representing a coherent and realistic development of the system along a specific trajectory defines a pathway.

In the FABLE Calculator, users define 16 parameters with multiple alternative values to represent scenarios. Implementing future scenarios in the model involves parameter shifters, which use historical values or past trajectories to capture time-specific relative changes in a parameter's initial value, allowing it to vary over time.

The model follows interconnected calculation steps, starting with estimating the agricultural food and non-food demand trajectory by 2050, modified by shifters reflecting population growth, GDP projections, diets, food waste, and biofuel demand. The model then considers various constraints tied to agricultural productivity, post-harvest losses, trade policies (e.g., import reduction and export quotas), and the availability and use of land. As a result of these assumed constraints, the model computes a feasible supply that is available for human consumption. The feasible supply might not align with the initially calculated supply target necessary to meet food demand when the constraints for expanding production are binding.

This feasible supply calculation is a key model output as it helps identify potential gaps between food demand and supply. It also provides insights into how much additional food is needed through production increase or imports to bridge these gaps.

As a critical component of the feasible supply, the feasible domestic food production plays a significant role, which is influenced by agricultural productivity and land availability. The model represents six land cover types and calculates land-use changes based on afforestation, reforestation, agricultural land expansion, and protected areas. A feedback loop ensures land-use trajectory assumptions remain within feasible restrictions concerning available land.

A main advantage of the FABLE model lies in its ability to address complex food and land-use system questions using a simplified setup. This user-friendly design enables researchers, policymakers, and stakeholders with diverse modeling expertise to operate the model using only basic Microsoft Excel knowledge and a moderate learning curve. Users can easily adapt the model by modifying scenarios, parameters, variables, and formulas. Its interactive interface supports transparent communication between modelers and stakeholders.

Although the model is user-friendly and requires minimal modeling expertise, it is important to note that the FABLE Calculator is an accounting model rather than an optimisation model. Consequently, it does not consider price effects and market dynamics. The options for reducing agricultural GHG emissions in the model rely on decreasing production volumes and enhancing productivity, while more sophisticated mitigation techniques, such as refined rice management or animal feed supplements, are not included in the model.

A more detailed explanation of the model and its application in different studies is available in the supplementary information.

### An empirical application to The Gambian food and land-use system

To analyse potential future pathways of the food and land-use system in The Gambia, we adapted the FABLE Calculator to better fit the country's context. We drew upon key parameters from previous FABLE modelling exercises (Mosnier et al., 2023) to inform the initial future scenarios for The Gambia's food and land-use system. To ensure the Gambia-specific parameter assumptions and scenario pathways were accurate and relevant for the Gambian food system, we organised a stakeholder workshop with over 30 participants, including technical experts, policymakers, researchers, farmer organisation representatives, and NGOs.

During the workshop, stakeholders examined the model's calculation steps, data used, parameter assumptions, and scenarios. They identified key drivers significantly influencing The Gambia's food and land-use system and collaborated in multidisciplinary breakout groups to co-develop and co-design realistic scenario pathways. Stakeholders reviewed initial model assumptions and results, providing feedback on drivers, relevant indicators, and additional national data and policy documents necessary for integrating these pathways into the FABLE Calculator.

### Parameters of the FABLE Gambia model

To estimate future domestic food demand in The Gambia, we used data on changes in population size and per capita food available for consumption. The latter is based on the current average daily per capita supply of different food groups, as documented in the FAO Balance sheets (FAO, 2023) (Fig. S1). Population projections consider alternative assumptions regarding future fertility, mortality, migration, and educational transitions that correspond to the five shared socioeconomic pathways (SSPs) (KC and Lutz, 2017) (Fig. S2). For the baseline scenario, we selected the intermediate scenario (SSP-2), which assumes an annual population growth of 2.8%, in line with The Gambia's growth rates in the 2010s, ranging between 2.5% and 3% (World Bank, 2023). The range of five SSP scenarios was used to account for the uncertainty in food demand stemming from varying population growth projections.

The future Gambian food supply scenarios were primarily determined by variations in domestic food production. This was influenced by scenarios considering the impacts of climate change and different field management practices on crop productivity. We applied these scenarios to the most widely grown and consumed crops in The Gambia, including rice, millet, maize, sorghum, groundnut, and cassava. Climate change scenarios, which account for the effects of temperature, rainfall, and other climate factors on crop yields, were derived from the Representative Concentration Pathway (RCP) 2.6 and RCP 6.0 scenarios. Our analysis considered crop yield changes due to climate change with and without CO_2_ fertilisation using LPJmL vegetation model simulations (Schaphoff et al., 2018) from the ISIMIP database (Arneth et al., 2017) (Fig. S3). As no climate projections were available for sorghum, we mapped the relative yield changes of millet, as it has similar biophysical and photosynthetic properties (Janssens et al., 2020).

We considered three field management scenarios that affect crop yields:

1. Business as usual (BAU) scenario: This scenario assumes no significant changes in agricultural practices or technologies and was used as a reference for comparison with other scenarios.
2. Climate change adaptation (CCA) scenarios: CCA practices typically respond to shifts in precipitation and temperature to enhance productivity. These practices include shifting planting dates, changing crop varieties (e.g., drought-resistant crops), crop rotations, altering soil management and fertilisation, and improving water management, among others (Segnon et al., 2021). The estimated impact of CCA practices on crop yields was based on a systematic review of climate change and adaptation strategies on major crops in West Africa (Carr et al., 2022), which showed that crop yields can increase by 13% on average by adopting techniques such as optimised planting dates, climate-resistant varieties, and increased fertiliser use under changing climate conditions.
3. Intensified nutrient and water management (Boost) scenarios: We estimated the increase in crop yield resulting from improved access to irrigation and fertiliser based on simulated yield potentials achievable with reduced water and nutrient stress. This agricultural intensification scenario assumes that with better management, 75% of The Gambia's yield potential can be achieved, based on yield potentials calculated by Mueller et al. (2012). Achieving these yields would require increased irrigation and nutrient application across most of the country, bringing crop yields closer to the respective yield potentials achieved in the world's main agricultural areas. The yield potentials were used as a target for the year 2050, which would be gradually reached through a linear growth function in 5-year increments. If the historical maximum yield already exceeds 75% of the achievable yield potential, the historical yield was used as the intensification target (Fig. S4).

To evaluate whether domestic crop production could meet the increasing food demand through productivity gains, we kept other factors affecting food supply constant from the baseline years 2000–2010, including cropland area, livestock production productivity, food waste, post-harvest losses, and export quantities. Additionally, our model assumes a constant import share, meaning that imports will represent the same share of the domestic supply in 2010 and in 2050. Table 1 summarises the parameters relevant to the FABLE Gambia model.

**Table 1:** Summary of parameters relevant to the FABLE Gambia model

**Parameter**	**Options**	**Description**
Population	Annual population growth of 2.8% (SSP-2)	Shared socioeconomic pathways (SSP) are used to estimate population growth based on future fertility, mortality, migration, education (KC and Lutz, 2017). The SSP-2 scenario served as a baseline, while the range from SSP-1 to SSP-5 captured various potential paths of population growth and associated changes in food demand
Diet	Current diet	Current dietary patterns were derived from the average daily per capita supply of different food groups for the years 2000–2010, as recorded in the FAO Food Balance Sheets (FAO, 2023)
Climate Change	- RCP 2.6 - RCP 6.0	Changes in temperature, precipitation, and other climate factors were based on two Representative Concentration Pathway (RCP) scenarios, RCP 2.6 and RCP 6.0. Their effects on crop yields were simulated using LPJmL, with and without CO_2_ fertilisation (Schaphoff et al., 2018). The data was sourced from the ISIMIP database (Arneth et al., 2017)
Field Management	- BAU - CCA - Boost	Field management scenarios assess the impact of various approaches to agricultural practices and technologies on crop yields. These scenarios include BAU, which assumed no significant changes in current practices; CCA, which explored the effects of climate change adaptation strategies informed by a systematic review (Carr et al., 2022); and Boost, which was based on yield potentials with intensified nutrient and water management, as calculated by Mueller et al. (2012)
Other	Constant from baseline	Other factors include cropland area, import shares, export quantities, livestock productivity, food waste, and post-harvest losses. Values for these factors were taken from the years 2000–2010, as recorded in the FAO Food Balance Sheets (FAO, 2023) and previous FABLE modelling exercises (Mosnier et al., 2020, 2023)

### Considerations on fixed dietary scenario in food demand projections

While our model accounts for population growth as a key determinant of future food demand, it assumes fixed dietary patterns. This is a noteworthy simplification, as food demand is not only determined by population size, but also by dynamic dietary habits. These habits can be influenced by several socio-economic and cultural factors, including GDP, the degree of urbanisation, household characteristics, education levels and consumer preferences, each affecting the demand for certain types of food.

The wide range of factors influencing dietary choices increases the complexity and uncertainty of scenarios for future food demand. Moreover, Ali et al. (2023) have shown that common socio-economic and cultural factors cannot explain the recent changes in diets across all food groups in The Gambia, thus highlighting the complexity involved in projecting future food demand. By focusing solely on the influence of population growth, we aim to provide a baseline scenario that illustrates the pressing challenges The Gambia will face even if dietary habits remain unchanged.

## Results

### Future food demand and requirements for production and imports in The Gambia

**Fig. 1:**
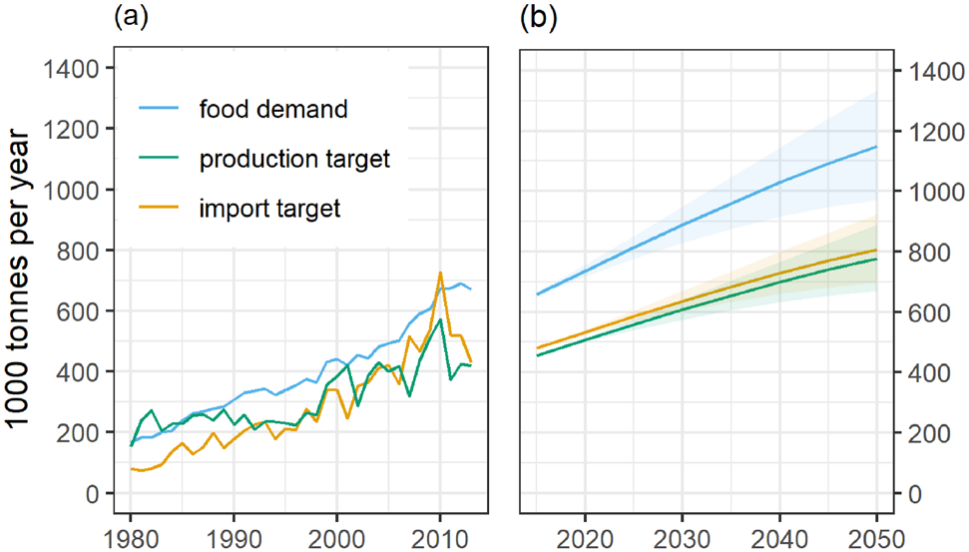
Food demand, food production and food imports in The Gambia as recorded by FAO (**a**) from 1980 to 2015 and projected using FABLE (**b**) from 2015 to 2050. Projected food production and food imports represent the target needed to meet food demand. Discrepancies between total food demand and the sum of production and import volumes arise due to factors such as exports, food waste and losses, unaccounted informal trade, stock variations, non-food uses and statistical discrepancies in FAO Food Balance Sheets. The projections include an uncertainty range due to the different SSP population growth scenarios represented by the shading. The projected change in food demand, production target, and import target for all major food groups can be seen in Figs. S5 to S7

Food demand in The Gambia is expected to increase under the baseline scenario by 54% by 2050, with variations ranging from 35 to 77%, depending on future population growth (i.e., SSP scenarios) (Fig. 1). The projected food demand growth is lower than the recorded historical growth, where between the 1990s and 2010s food demand in The Gambia grew by 106%, mainly due to population growth. During the same period, food production increased by 93%, and imports expanded by 160%. If this trend continues, rising food demand will be met with a greater increase in imports than in production.

**Fig. 2:**
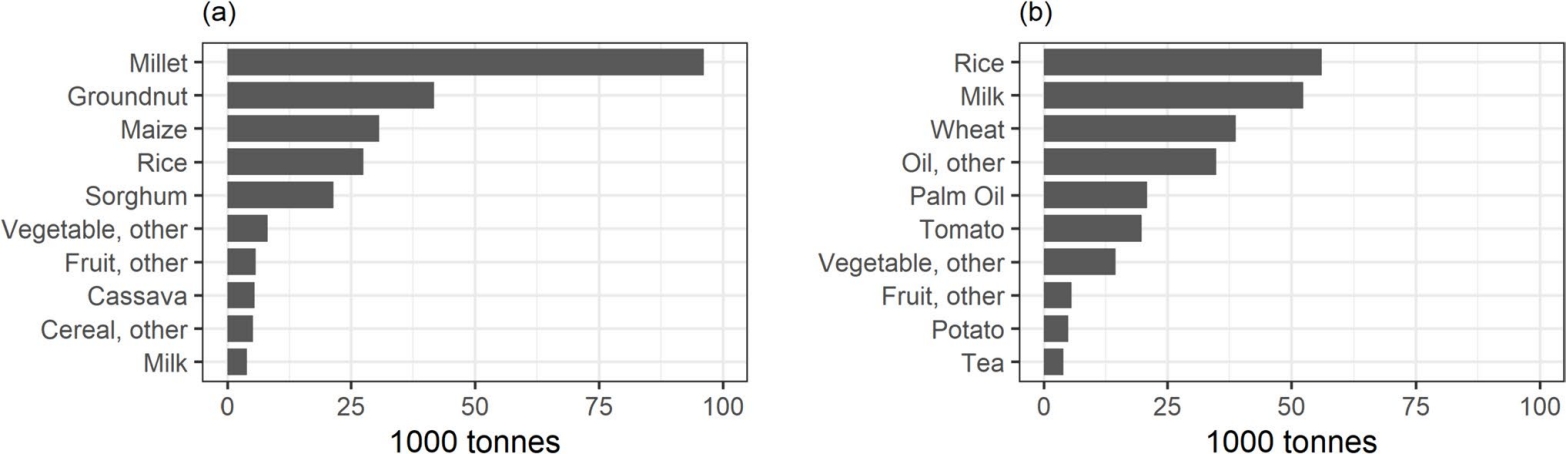
**a** Production growth required to meet food demand between 2020 and 2050 for the ten food products with the highest projected absolute increase. **b** Increase in imports required to meet food demand between 2020 and 2050 for the ten food products with the highest projected absolute increase

To meet the rising food demand in 2050, total domestic food production will need to increase accordingly. Assuming the area devoted to particular crops remains unchanged, the largest absolute increase in production will be for crops that are predominantly produced in The Gambia. This includes millet, groundnut, maize, and rice (Fig. 2(a)).

Import volumes will also need to increase to meet the projected food demand. Assuming constant import shares for consumed products, the largest increase in import volumes in absolute terms is for historically high-imported food groups, such as rice, milk, and wheat (Fig. 2(b)).

### Feasible food production under different climate change and crop productivity scenarios

Through our analysis of future food demand, as presented in Section 3.1, we identified a supply target required to meet projected demand through an increase in domestic food production and imports. However, inherent constraints in production and trade, such as land availability, land-use policies, and trade policies, can limit the feasible supply, causing it to fall short of the target.

As food demand in The Gambia grows, primarily due to population increase, the gap between demand and feasible supply increases under the business-as-usual scenario. This divergence is influenced by changes in feasible domestic food production, which is itself influenced by the impact of climate change on crops and field management practices.

**Fig. 3:**
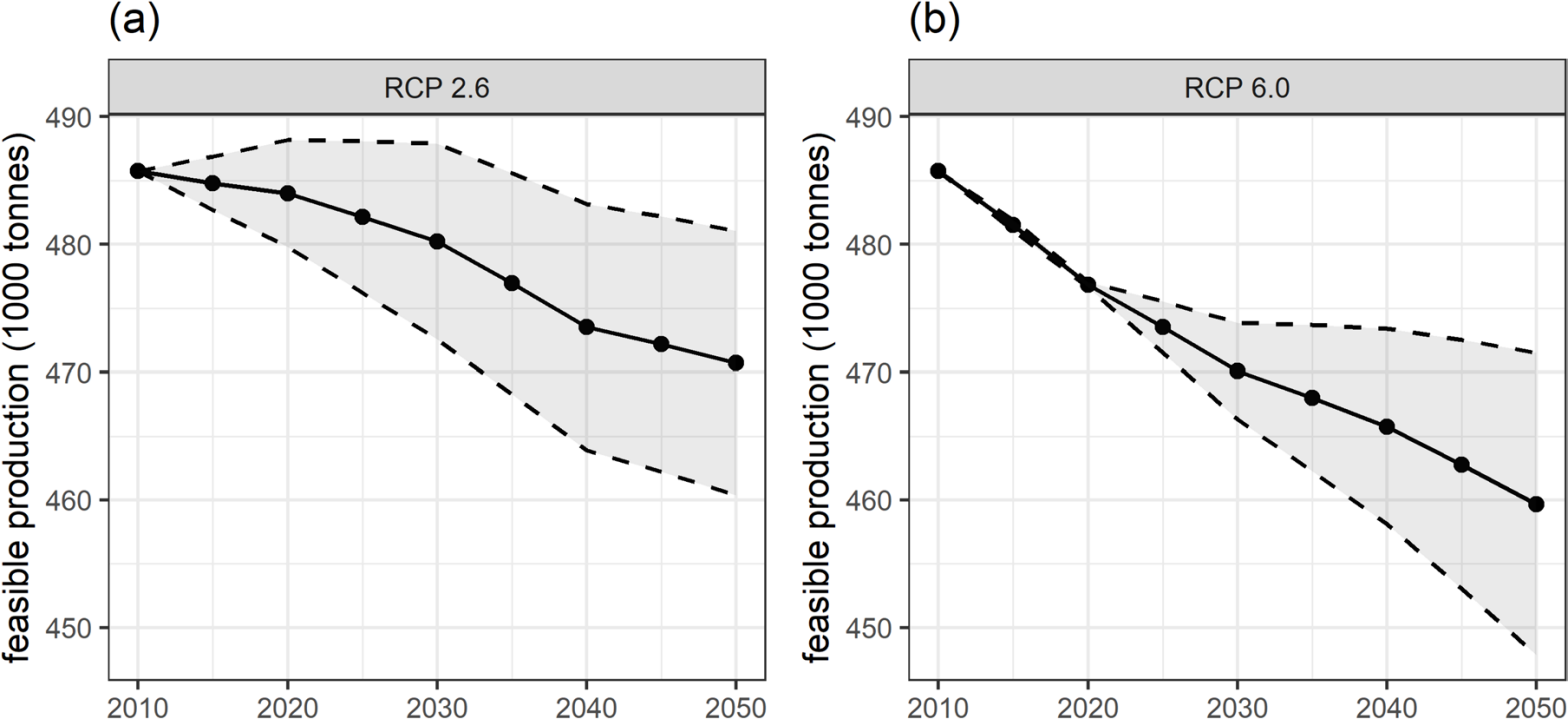
Feasible food production in The Gambia from 2010 to 2050 under business-as-usual field management under the RCP scenarios RCP 2.6 (**a**) and RCP 6.0 (**b**). Each trend line represents the average projected impact of climate change on food production. The shaded area indicates the uncertainty ranges due to the productivity effect of increased levels of CO_2_ (carbon fertilisation)

Feasible domestic food production is projected to decrease due to climate change under the business-as-usual field management (Fig. 3). By 2050, feasible food production is estimated to decrease by 3% under the RCP 2.6 scenario and 5% under the RCP 6.0 scenario. While the additional CO_2_ present in the atmosphere could have a so-called fertilisation effect on crop productivity and could potentially mitigate these declines, its impact is uncertain due to crop-specific variability and the difficulties in simulating this process. Accounting for this uncertainty, the impact could range from 1 to 5% under RCP 2.6 and from 2 to 8% under RCP 6.0.

**Fig. 4:**
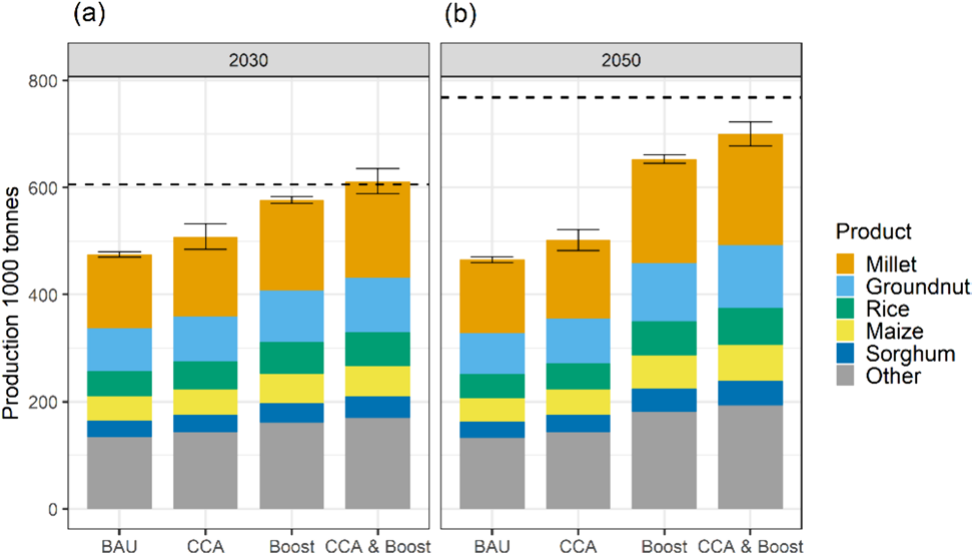
Feasible food production for domestic consumption and exports on current cropland under different crop productivity scenarios in 2030 (**a**) and 2050 (**b**). Crop productivity scenarios include business-as-usual (BAU), climate change adaptation (CCA), agricultural intensification (Boost), and a combination of both (CCA & Boost). Error bars indicate the variation between food production simulated under the RCP2.6 scenario and simulated under the RCP 6.0 scenario. The dashed line represents the target of domestic food production required to meet food demand for both domestic consumption and exports. The coloured products include the most produced food products in The Gambia. The projected changes in feasible food production for all major food groups can be seen in Fig. S8

In terms of food production, the differential impact of varying climate change scenarios is relatively minor when compared to the changes brought by improved field management. Figure 4 illustrates the variation in feasible domestic food production over time and under different scenarios, with differences in bars representing projected production changes due to field management and error bars indicating the different projected effects of climate change between the RCP 2.6 and RCP 6.0 scenarios.

The primary driver of changes in feasible food production are improvements in field management practices leading to increased crop yields beyond those in the business-as-usual (BAU) field management scenario. Climate change adaptation techniques (CCA) contribute to a 7% increase in feasible production in 2030 and an 8% increase in 2050 compared to the BAU scenario. Intensified fertiliser application and irrigation (Boost) results in a 21% production increase in 2030 and a 40% increase in 2050. Combining both approaches yields a 29% production increase in 2030 and a 51% increase in 2050. The larger combined effect occurs because CCA and Boost benefits are applied as multipliers in sequence, each increasing the already enhanced yields, rather than just adding the individual effects. The most notable improvements in feasible production are observed in crops with the highest current production levels, such as cereals (millet, rice, maize, sorghum) and groundnuts.

**Fig. 5:**
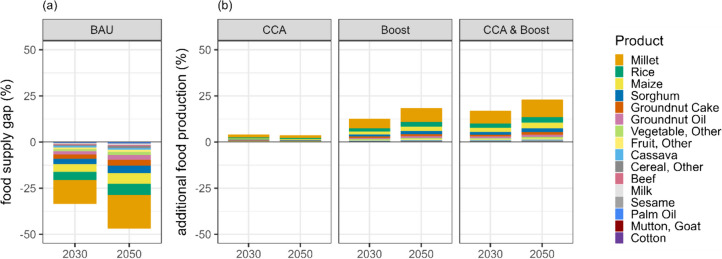
**a** Shortfall of food supply for domestic consumption (%), expressed as the gap between the food production target to meet food demand and the feasible food production in 2030 and 2050 under the business-as-usual scenario. **b** Additional food production on current cropland (%) estimated under different scenarios to increase crop productivity, including climate change adaptation (CCA), agricultural intensification (Boost), and a combination of both (CCA & Boost)

Domestic food production, even under all options to increase productivity, falls short of meeting the 2050 food demand target, resulting in supply gaps for various food and feed products (Fig. 5). Under the BAU scenario, the food supply gap for all food groups combined is projected to be 361,000 tonnes (47%) by 2050. Increasing productivity by adopting climate change adaptation and investing in agricultural intensification can help mitigate these supply gaps. Climate change adaptation practices can contribute an additional 28,000 tonnes (4%) of food production, while agricultural intensification can provide 141,000 tonnes (18%) of extra production. Implementing both approaches can generate an additional 176,000 tonnes (23%) of food production, which can approximately halve the missing food supply by 2050.

The projected gap between domestic demand and feasible domestic supply will lead to an increasing deficit in the food available for consumption by 2050. Under current dietary patterns, this would reduce the daily per capita calorie availability of 2553 by between 286 and 623 cal, depending on agricultural management practices (Fig. S9).

**Fig. 6:**
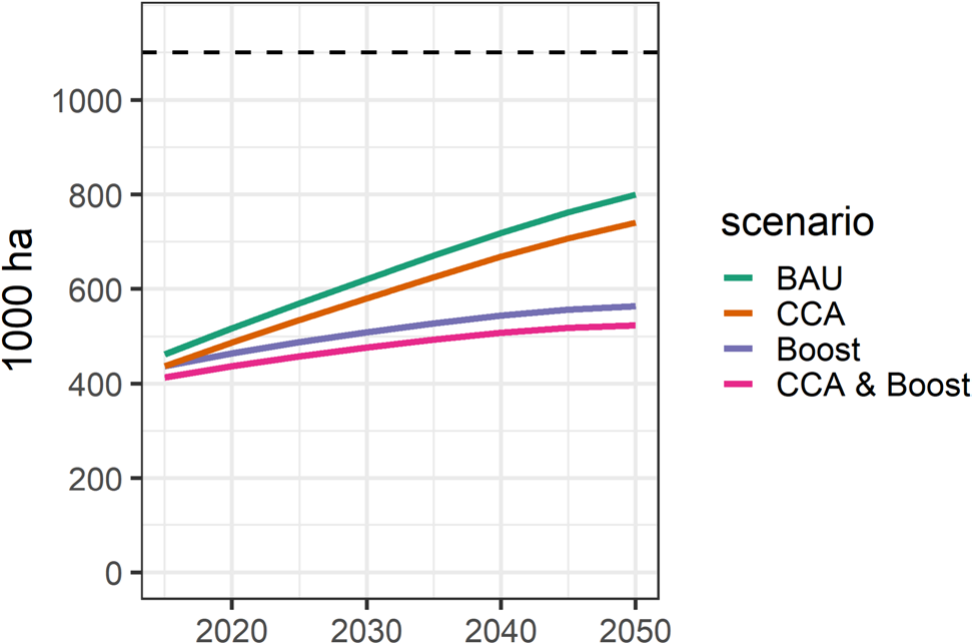
Change in the area of cultivated land required to meet food demand under different crop productivity scenarios from 2015–2050. Crop productivity scenarios include business-as-usual (BAU), climate change adaptation (CCA), agricultural intensification (Boost), and a combination of both (CCA & Boost). The dashed line represents the total area of The Gambia

To close the gap between food demand and supply, The Gambia would need to increase food imports, expand domestic cropland, or repurpose existing cropland from export-oriented crops to crops that serve domestic food consumption. To meet the required levels of food supply without increasing imports, total cropland area would need to increase by approximately 123% (431,000 ha) under the business-as-usual scenario and by about 56% (195.000 ha) under maximum crop productivity, compared to the baseline harvested area of 350,000 hectares (Fig. 6). Such land use alterations would affect deforestation, biodiversity, water resources, and greenhouse gas emissions. Our projections indicate that greenhouse gas emissions from crop production in 2050 could vary between 0.4 Mt CO2e under the business-as-usual scenario to 0.15 Mt CO2e under conditions of maximum crop productivity.

## Discussion

### Summary of findings

If recent trends continue, food demand in The Gambia will increase in the future to feed a growing population. To ensure that this growing demand can be met, it is crucial to implement strategies aimed at increasing food supply, which include increasing domestic production, increasing imports (and/or reducing exports), or a combination of these approaches.

In The Gambia, improving crop yields presents an opportunity to bridge the demand–supply gap. Our results indicate that if domestic yields of the most consumed crops would reach their potential achievable levels, the projected food supply gap in 2050 could be halved.

Whilst this is a promising improvement, additional measures would be required in The Gambia to fully close the supply gap, such as expanding existing agricultural land, repurposing cropland used for producing export and cash crops to producing domestically consumed food, or modifying trade patterns (i.e., increasing imports and/or decreasing exports).

Adopting climate change adaptation technologies has a limited impact on increasing yield potentials in The Gambia compared to boosting productivity. This is fundamental as the main determinants of low productivity are related to limited access to inputs and technology rather than climate-change-induced yield fluctuations. Nevertheless, climate change adaptation remains crucial for food security, as this helps mitigate the risk of major harvest failures resulting from extreme events such as droughts.

Our findings echo previous studies that emphasised the importance of supporting domestic agricultural production in sub-Saharan Africa to increase self-sufficiency in food supply amid rising demand (De Graaff et al., 2011; Giller, 2020; Luan et al., 2013).

### The role of diets in The Gambia's changing food demand

Demand projections for specific food groups in The Gambia carry considerable uncertainty due to evolving dietary habits, which can be influenced by a variety of factors such as socio-economic changes and government policies (Ali et al., 2023). Currently, cereals like rice and wheat dominate food consumption, while healthier options such as fruits, vegetables, and nuts are not adequately consumed (Ali et al., 2022).

To address unhealthy diets, the Gambian government proposed the Food-Based Dietary Guidelines (FBDG) to educate the population on healthier eating habits. If healthier diets would be adopted, this would considerably shift the food demand projections and consequently the projected supply–demand gap. Specifically, the supply gap for cereals would decrease, while the gap for fruits, vegetables, pulses, nuts, and meat would increase.

Beyond dietary guidelines, broader socio-economic indicators like GDP growth and urbanisation also have the potential to influence a shift in diets, likely leading to an increase in demand for animal-source foods in The Gambia (Ali et al., 2023).

Gambian farmers and producers may face challenges from these changes, especially for fruits and vegetables. These healthier options typically require intensive use of resources such as fertiliser and water, advanced technology, and agricultural expertise. Currently, these resources are predominantly allocated to cereal production. In addition, the supply chain for shifting to more fruits and vegetables would require investment in infrastructure for perishable food.

Addressing the supply challenges will require strategies that improve trade flows and promote domestic production of nutrient-rich crops. Even without substantial changes in dietary patterns, major supply gaps are projected for the coming decades. The next section will discuss implications and potential solutions related to domestic production and international trade.

### Policy and practice recommendations

#### Enhancing agricultural productivity in The Gambia

The current agricultural productivity in The Gambia is significantly lower than that of neighbouring countries, with cereal yields 42% and 35% lower than in Senegal and the average for West Africa in the past decade, respectively (FAO, 2023). Among the cereals, rice has the greatest potential for yield improvement, with achievable regional average yields being up to five times greater than recent average Gambian yields. In addition, root and tuber crops such as cassava have enormous untapped potential to increase yields by five to ten times their current levels and have the added advantage of being highly resilient to climate change (Jarvis et al., 2012).

A major barrier to higher crop production is the inadequate access of farm households to inputs such as fertilisers, seeds, and extension services (Gajigo & Saine, 2011). Furthermore, poor land management practices and land degradation have contributed to declining soil fertility and crop productivity, as evidenced by regional satellite data (Mechiche-Alami & Abdi, 2020).

Given the documented benefits of public agricultural spending for food security (Bambio et al., 2022), prior studies have recommended increasing agricultural investment in The Gambia (Belford et al., 2023; Fontan Sers & Mughal, 2020). Although the government subsidises a large proportion of the fertiliser used in the country, its usage remains lower than in neighbouring Senegal and the West African average (World Bank, 2019). The high costs of fertiliser contribute to this low usage, as it renders fertiliser inaccessible for smallholder farmers, even when it is available (Segnon et al., 2022a, b).

Enhancing productivity requires increased access to inputs such as pesticides and fertilisers, advanced technologies like new seed varieties, and improved water management practices (Government of The Gambia, 2019; World Bank, 2019). Investment in rice production has shown promising results for increasing productivity in The Gambia. For example, The Rice Value Chain Transformation Programme has increased access to improved seeds, fertilisers, and machinery, resulting in higher rice yields (Belford et al., 2023). However, increasing productivity does not always require significant investments in new inputs or technology. Field trials of the System of Rice Intensification (SRI) have demonstrated that improved agricultural practices can boost productivity without relying solely on water and fertilisers (Ceesay, 2011). This underscores the importance of providing farmers with information and resources to improve farming practices, in addition to investments in inputs and technology.

Adopting innovative, land-efficient farming practices and the use of underutilised rural land can increase food production without the need for large-scale cropland expansion (Ali et al., 2022). Urban farming methods such as vertical farming allow for optimal use of space, while homestead farming enhances production even in confined spaces. Collective farming initiatives, such as community gardens, not only increase local production but also promote community empowerment, with women playing a central role (Bizikova et al., 2020; Ebile et al., 2022). These approaches offer opportunities to increase the production of fruits and vegetables in particular. However, as fruits and vegetables are highly perishable, it is essential to invest in post-harvest infrastructure to reduce losses from storage and transport.

Given The Gambia’s vulnerability to climate change impacts, scaling up adaptation practices in agriculture and food systems is key to increase resilience to climatic risks. Several field studies in The Gambia and West Africa have confirmed the positive impact of various climate-smart agriculture techniques in mitigating climate change effects (Segnon et al., 2022a, b; Zougmoré et al., 2014, 2018). Priority adaptation options, determined through evidence-based assessment and participatory processes (Segnon et al., 2022a, b), offer a strategic pathway for the expansion of these practices.

For example, climate change-induced weather variability necessitates reliable weather information for agricultural planning, helping farmers to make informed decisions and reduce risks associated with weather-related crop failures (MacCarthy et al., 2017; Segnon et al., 2022a, b). Additionally, cultivating crops better adapted to tropical climates can be a promising strategy for ensuring a stable future food supply. Duvallet et al. (2021) found that native crops like sorghum, millet, and tubers are more resilient to climate variability and have higher yields compared to rice in West Africa. By increasing the production of such local crops, The Gambia could secure a more stable, climate-resilient food supply.

#### Enhancing food supply in The Gambia through trade

Enhancing agricultural productivity in The Gambia was projected to reduce The Gambia’s dependence on food imports in our model. However, as results show, the growing demand could not be fully met with increasing domestic production, thus an increase in food imports would likely be necessary. Currently, the most consumed cereals in the Gambia (largely rice and wheat) are supplied mostly through imports, while the consumption of locally produced non-import-reliant foods, like millet, has declined. A shift to more diverse diets could result in greater reliance on imports for a number of food groups that are currently not produced in sufficient quantities domestically, like fruits and vegetables.

Opening up trade can enhance dietary diversity by making a more diverse range of food groups available, thus contributing to improved diet quality (Dithmer & Abdulai, 2017; Kinnunen et al., 2020). Trade plays an important role in ensuring consistent access to food by fostering better connections between food-deficit regions and those with surpluses (Janssens et al., 2020). Ensuring smooth trade flows, particularly for perishable foods, is therefore essential for building a resilient food system.

However, import-dependent countries like The Gambia must be prepared for potential disruptions in international trade due to tariffs, trade restrictions, or global events. Recent examples include the 2007–2008 global food crisis, the COVID-19 pandemic, and the war in Ukraine, a major exporter of agricultural products (Ali et al., 2020; FAO, 2022; Janssens et al., 2020; Sperling et al., 2022). Climate change could also contribute to future shocks in food trade, highlighting the importance of considering the climate resilience of trading partners (Gaupp et al., 2020; Hadida et al., 2022). Diversifying trade partners can spread the risk of supply disruptions and allows for more flexible adjustments in case of climate shocks or export difficulties in a specific country or region.

Additionally, enhancing regional trade can reduce reliance on global market fluctuations and help mitigate food supply risks. Geographically closer trade partners offer reduced transportation time and costs, ensuring that fresher products can reach consumers quickly, which is vital for perishable items. Initiatives like the African Continental Free Trade Agreement could help to integrate Africa's food markets by promoting policy harmonisation, trade barrier reduction, and smooth cross-border movement of goods, which can contribute to stable prices and consistent access to various food items (Morsy et al., 2021).

To improve trade within the Economic Community of West African States (ECOWAS) region and maximise its positive impact on The Gambia's access to food, it is essential to address several trade barriers. Currently, barriers such as inadequate funding on both soft (e.g., inspectors, customs digitisation) and hard infrastructure (e.g., roads, ports), and informal cross-border food trade undermine trade competitiveness and revenue collection (Kareem & Wieck, 2021). Intra-African trade is low compared to other continents, and trade between regional economic communities remains limited (Janssens et al., 2022). Although The Gambia has regional trade partners like Senegal, its largest trade partners are currently in South America, Europe, and Asia (Hadida et al., 2022). Overcoming these barriers and strengthening regional trade relationships could contribute to a more coherent and resilient food supply in future.

### Opportunities and limitations in modelling future food supply and demand in The Gambia

Our analysis of future food supply and demand scenarios in The Gambia made use of the compatibility of FABLE with common food system databases to mitigate low local data availability. Yet, the complexity of food systems and limited data introduce some limitations.

Data mainly stems from FAO food balance sheets, known for potential inaccuracies such as underrepresentation of fruit and vegetable consumption (Ali et al., 2022; Del Gobbo et al., 2015). These may lead to inaccuracies in demand projections. Moreover, changes in future diets were not considered in our projections. In addition, our study does not consider subnational differences in population growth and diets. A regional analysis may reveal significant disparities in food availability, reflecting increased undernutrition in rural areas (Government of The Gambia, 2019). Incomplete trade data due to informal trade, scarce livestock and fisheries data, and unavailable food waste or post-harvest loss data, further restrict model comprehensiveness.

Diverse field management in The Gambia contributes to variable future crop productivity potentials, which are compounded by regional differences in farming techniques and adoption of climate change adaptation methods. To address this, we applied business-as-usual and two further scenarios assuming widespread climate change adaptation and agricultural intensification. Although not exhaustive, this approach captures a broad range of potential productivity outcomes.

Despite its limitations, this study’s modelling framework provides essential insights into The Gambia’s various future food supply and demand scenarios. Additionally, it offers a foundation for further research into the feasibility of healthy dietary targets and associated food availability.

## Conclusion

Our analysis of different future food demand and supply scenarios in The Gambia reveals that the country's food security faces ongoing challenges. The projected increase in food demand will likely lead to a growing reliance on imports, particularly if healthier and more diverse diets are adopted in the future. Nonetheless, The Gambia has significant potential to improve agricultural productivity, closing the considerable gap between actual and achievable yields.

To boost agricultural productivity in The Gambia, recommendations include targeted investments in research to increase yields and the implementation of subsidy programmes to facilitate smallholder farmers' access to key inputs such as fertilisers and seeds. Alongside this, the development of sustainable irrigation infrastructure and the cultivation of unused rural land can enrich the food supply. Expanding agricultural extension services can facilitate the spread of innovative farming practices that make efficient use of land. It is also important to allocate resources to modern post-harvest infrastructures to minimise food loss, especially for perishable products. Developing climate-adapted agricultural techniques, tailored to the local conditions through evidence-based assessment and participatory processes, can help farmers adapt to climate variability. In addition, improved weather forecasting services could mitigate the risks of weather-related crop failures.

In terms of trade, diversification of trading partners can reduce risks related to supply chain disruptions and market volatility. In addition, strengthening regional trade within ECOWAS can reduce exposure to global market volatility, lower transport costs and ensure faster access to fresh produce. This requires addressing deficiencies in soft and hard infrastructure as well as informal cross-border food trade to improve trade competitiveness and revenue collection.

These general policy starting points serve as a basis for more detailed future research on policy implications. Additionally, investigating the feasibility of the strategies we have outlined to improve agricultural productivity could provide key insights into increasing The Gambia's food supply. Given the dynamic and multi-layered nature of food systems, ongoing research is essential to fully understand the feasibility and variability of food supply and demand in the country. Our modelling framework, based on open-access data and scenarios, provides a starting point for much needed future research on food security in The Gambia and other countries facing similar challenges.

### Supplementary Information

Below is the link to the electronic supplementary material.

Supplementary file1 (DOCX 823 KB)

## Data Availability

The results that support the findings of the study are provided in the manuscript and supplementary material. Scenario results for all scenarios are made accessible online via a Rshiny app: https://gambia.shinyapps.io/FABLE-Gambia/. Additional data and code are available upon reasonable request from the corresponding authors. Source data are provided with this paper.

## Footnotes

1 An Open-Source version of the FABLE Calculator can be downloaded for free at https://www.abstract-landscapes.com/fable-calculator
