# Supplementary material for: Addressing future food demand in The Gambia: can increased crop productivity and climate change adaptation close the supply–demand gap?
Source: Food Secur. 2024 Apr 26;16(3):691–704. doi: 10.1007/s12571-024-01444-1 (PMC11102352; doi:10.1007/s12571-024-01444-1)
Supplement: Supplementary file 1 — Supplementary file1 (DOCX 823 KB) [file 12571_2024_1444_MOESM1_ESM.docx]

# Supplementary Information

**Text S1: The FABLE Calculator**

**Model Description:**

The FABLE Calculator^[[1]](#footnote-1)^ developed by Mosnier et al. (2020) is an Open-Source Excel accounting model that studies the potential evolution of food and land-use systems from 2000 to 2050 and assumes agriculture as the main driver of land-use change. Using historical FAOSTAT data (from 2000-2010) as input data, the model approximates from 2015 onward in five-year time steps the agricultural demand and supply and potential evolution of the food and land-use sectors by modeling drivers related to food and land-use systems through a combination of scenarios. The impacts of the selected scenarios on future food security, production, trade, land use, land use change, greenhouse gas emissions, biodiversity, and water footprint are assessed.

**Model Applications:**

The FABLE calculator has been applied and adapted to 22 country teams within the FABLE Consortium^[[2]](#footnote-2)^, with four broad objectives.

1. National-level integrated scenario analysis of food and land-use systems, as conducted in Mexico, India, the United States, and Canada (González-Abraham et al., 2023; Jha et al., 2023; Wu et al., 2023; Zerriffi et al., 2023). These analyses focused on exploring synergies and trade-offs between food systems and other sectors, such as Mexico's efforts to achieve self-sufficiency in its food system while meeting mid-century climate, conservation, and production goals in the land-use sector.
2. Evaluating sustainability implications of current long-term development policies, such as Rwanda's Vision 2050 plan (Perez-Guzman et al., 2023). This study highlighted the trade-offs between providing necessary nutrients for the Rwandan population and current agricultural practices and policies.
3. Investigating the impact of alternative agricultural production forms, as demonstrated in the study of organic agriculture in Sweden (Basnet et al., 2023). The research found that a future sustainable agriculture sector could be achieved through organic agriculture, but only when combined with other food system measures, such as changes in consumption patterns, more sustainable agricultural practices, and decreased food waste.
4. Co-developing scenarios through stakeholder engagement for training and policy discussions around food systems, as exemplified by Rasche et al. (2023) and Smith et al. (2023). Rasche et al. (2023) applied the model to co-develop scenarios with stakeholders, focusing on integrated modeling training and food policy discussions. In a similar way, Smith et al. (2023) co-created three pathways exploring UK land use strategies for addressing climate change, biodiversity loss, and sustainable development, engaging key stakeholders from recognized UK institutions.

**Modeling Scenarios and Calculation Steps:**

Assessing future trajectories of the model requires modifications to the drivers of system change, which depend on the choice and combination of model parameters and assumptions (i.e., scenarios). In this regard, user-defined combinations of scenarios, which represent coherent and realistic development of a system along a specific trajectory, define a pathway. The FABLE model allows users to define 16 parameters with multiple alternative values that can be adjusted to represent a scenario^[[3]](#footnote-3)^. A key concept for implementing future scenarios is the use of parameter shifters, based on either specific historical values or past trajectories. These shifters capture time-specific relative changes applied to a parameter's initial value, enabling the parameter to vary over time.

The model consists of several interconnected calculation steps. The starting point is to calculate the agricultural food and non-food demand trajectory by 2050 based on historical values of food demand and supply. These values are modified through shifters that capture scenario selection assumptions on population growth dynamics, GDP projections, diets, food waste, and biofuel demand^[[4]](#footnote-4)^. The feasibility of the agricultural sector to supply the targeted demand depends on assumptions on crop and livestock productivity, climate change, post-harvest, and trade (i.e., imports and exports).

Land availability plays a crucial role in determining the feasibility and self-sufficiency of agricultural production. The model represents six land cover types: cropland, pasture, forests, new forests, urban, and other land, which accounts for the difference between the previous categories and the total national land availability. Calculated land-use changes are influenced by assumptions regarding afforestation or reforestation, productive and urban land expansion, and protected areas. This is particularly important as it establishes a feedback loop ensuring that land-use trajectory assumptions remain within feasible restrictions concerning available land. For example, a country cannot expand its agricultural production beyond its borders or the allowable area, given the evolution of other land-use changes. Consequently, while crop expansion is possible, it cannot exceed available land resources and the policies or assumptions on other land uses. Any discrepancy between the targeted supply and land limitations (based on demand assumptions) is readjusted to calculate a feasible supply, given the constraints.

**Advantages and Disadvantages of the FABLE Model:**

A main advantage of the FABLE model lies in its ability to address complex food and land-use system questions using a simplified setup. This user-friendly design enables researchers, policymakers, and stakeholders with diverse modeling expertise to operate the model using only basic Microsoft Excel knowledge and a moderate learning curve. Users can readily adapt the model by modifying scenarios, parameter assumptions, variables, and formulas or by defining new modules. Furthermore, global datasets can be tailored to regional or country-level data to examine specific policies and scenarios. The model's interactive interface, particularly for scenario selection and adjustments, fosters straightforward and transparent communication between modelers and stakeholders.

Although the model is user-friendly and requires minimal modeling expertise, it is important to note that the FABLE Calculator is an accounting model rather than an optimization model. Consequently, it does not account for price effects and market dynamics. The options for reducing agricultural GHG emissions in the model rely on decreasing production volumes and enhancing productivity, while more sophisticated mitigation techniques, such as refined rice management or animal feed supplements, are not included in the model.


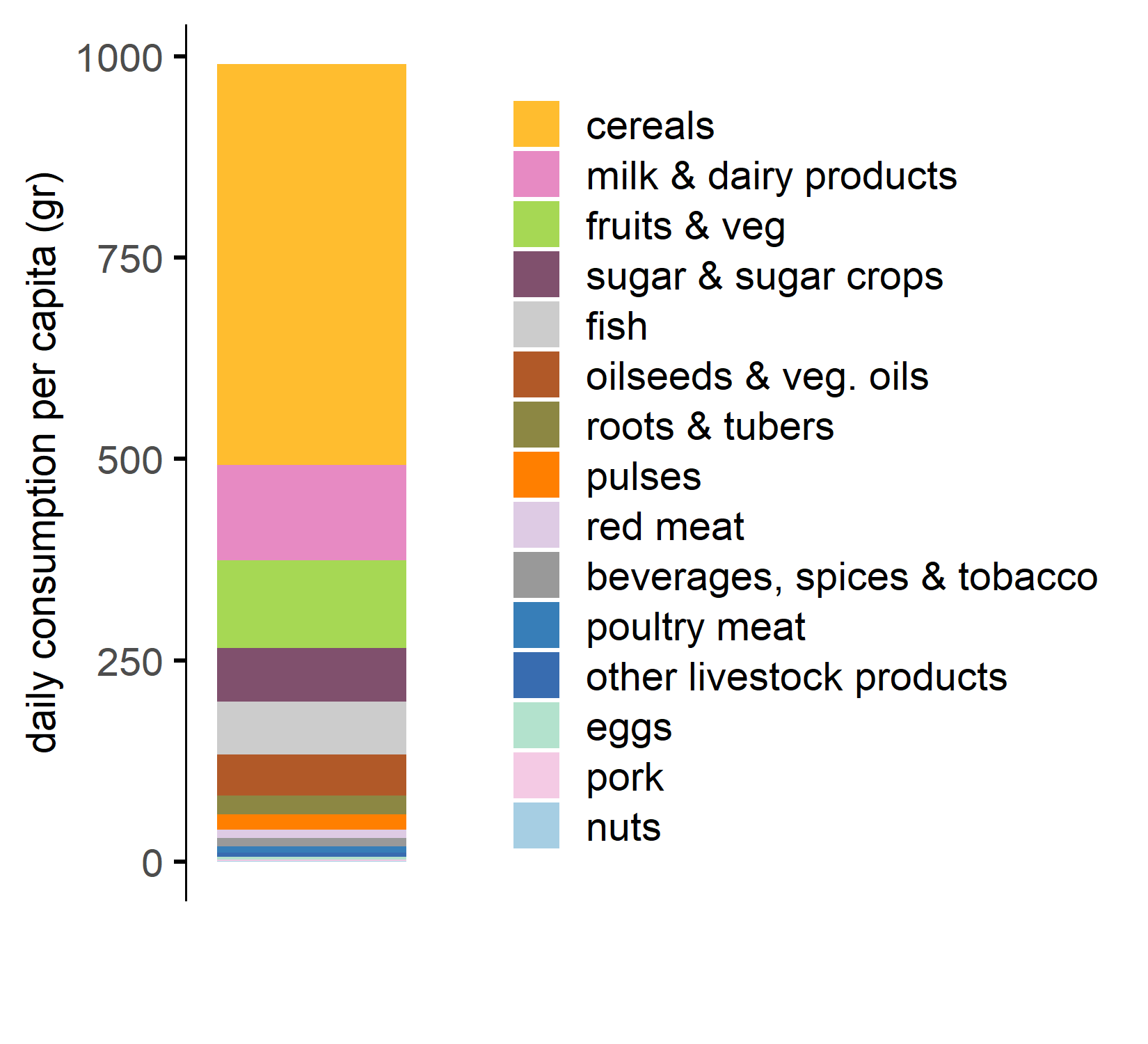


Figure S 1: Average daily per capita supply per food group (in grams) for the baseline years 2000 - 2010 (FAO, 2023).


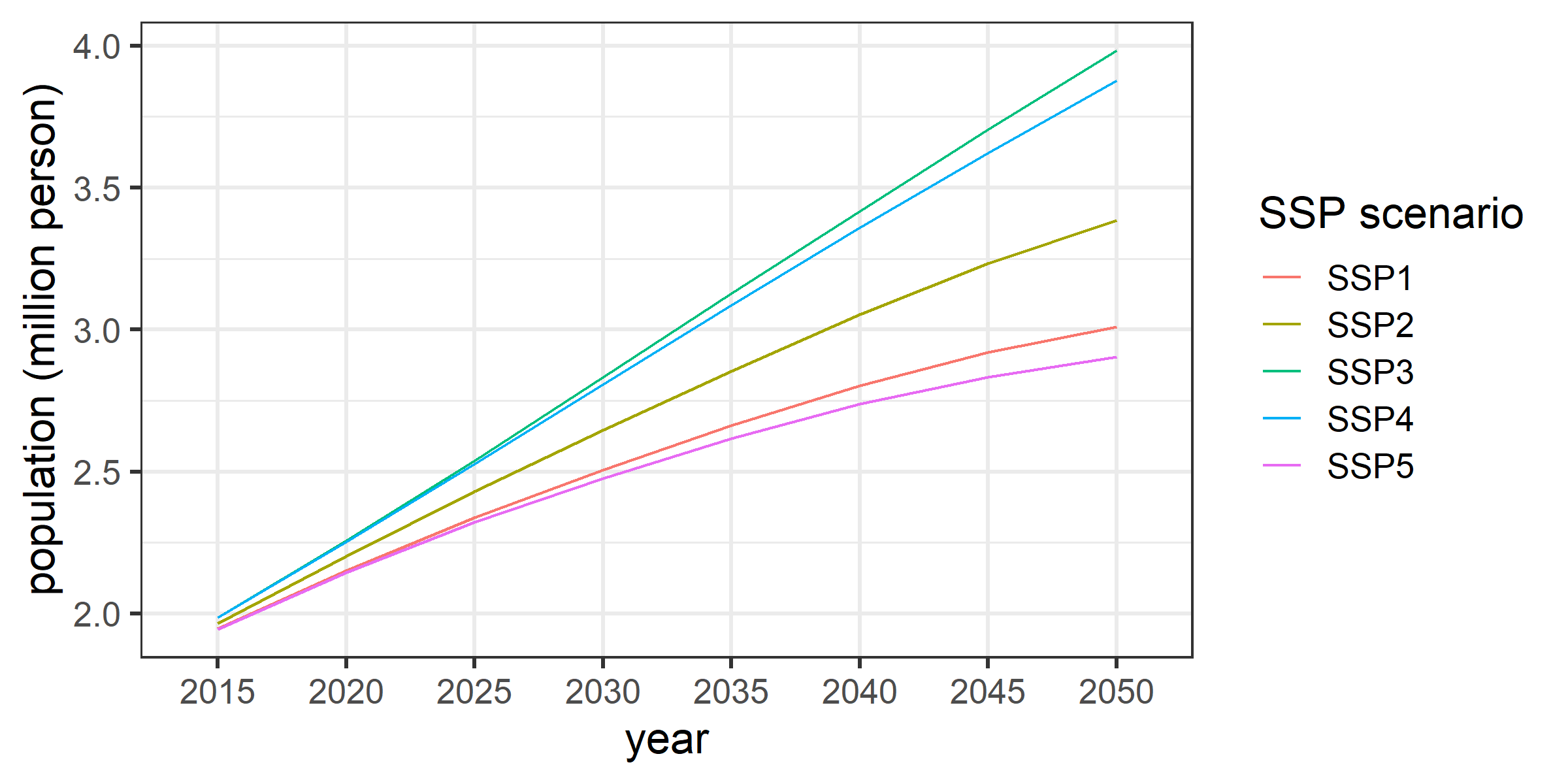


Figure S 2: Estimated population development in The Gambia based on the shared socioeconomic pathways (SSP) 1 to SSP 5 for estimating population growth based on future fertility, mortality, migration, and education (KC & Lutz, 2017).


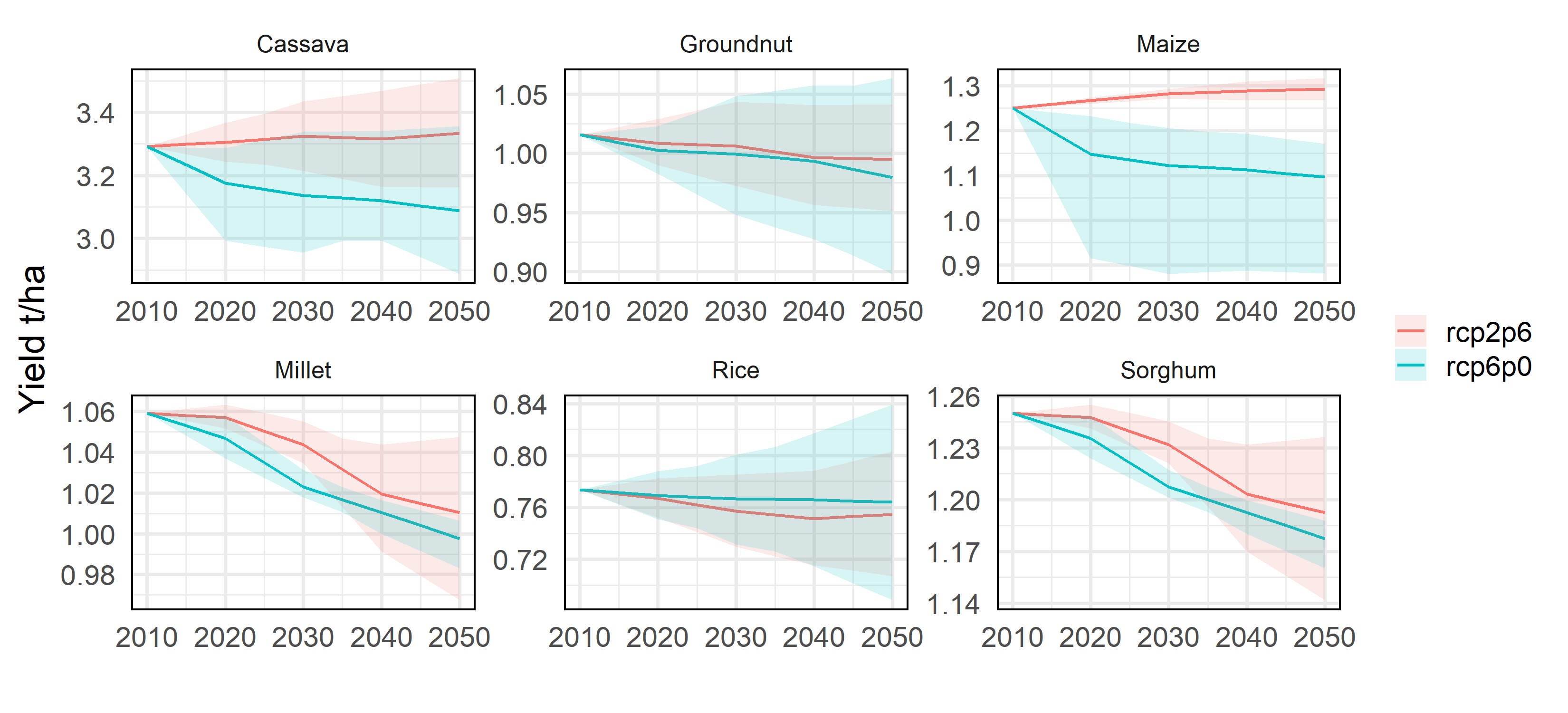


Figure S 3: Estimated average yield changes of the most widely grown crops in The Gambia due to climate change based on the Representative Concentration Pathway (RCP) 2.6 and RCP 6.0. Shadows illustrate the uncertainty range of the crop yield changes simulated with and without CO2 fertilisation and different climate models (Arneth et al., 2017).


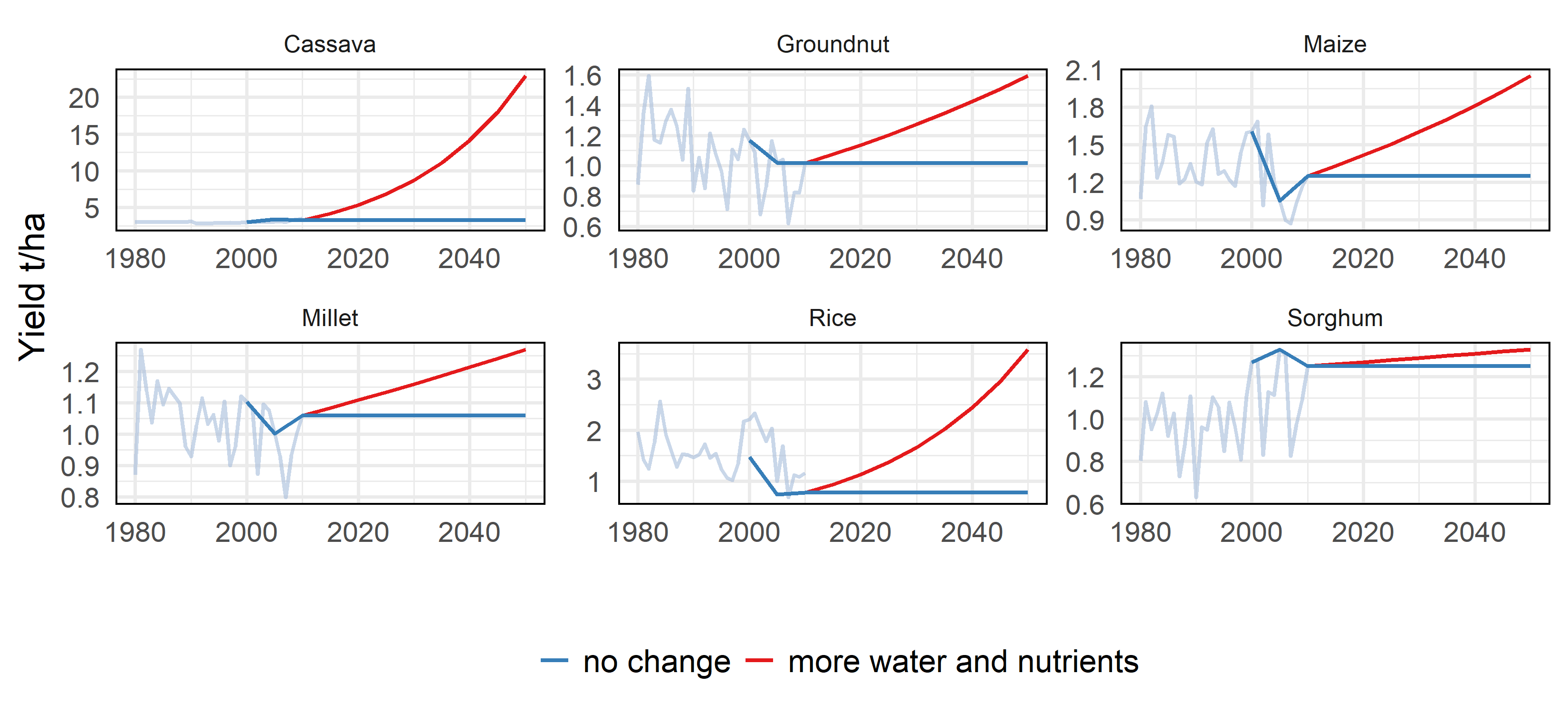


Figure S 4: Estimated yield changes of the most widely grown crops in The Gambia simulated with and without intensified nutrient and water management (Boost scenario). Yield changes under intensified management are targeted to reach 75 % of the achievable yield potential by 2050. (Mueller et al., 2012). If the historical maximum yield surpasses the 2050 achievable potential, it is used as the intensification target.


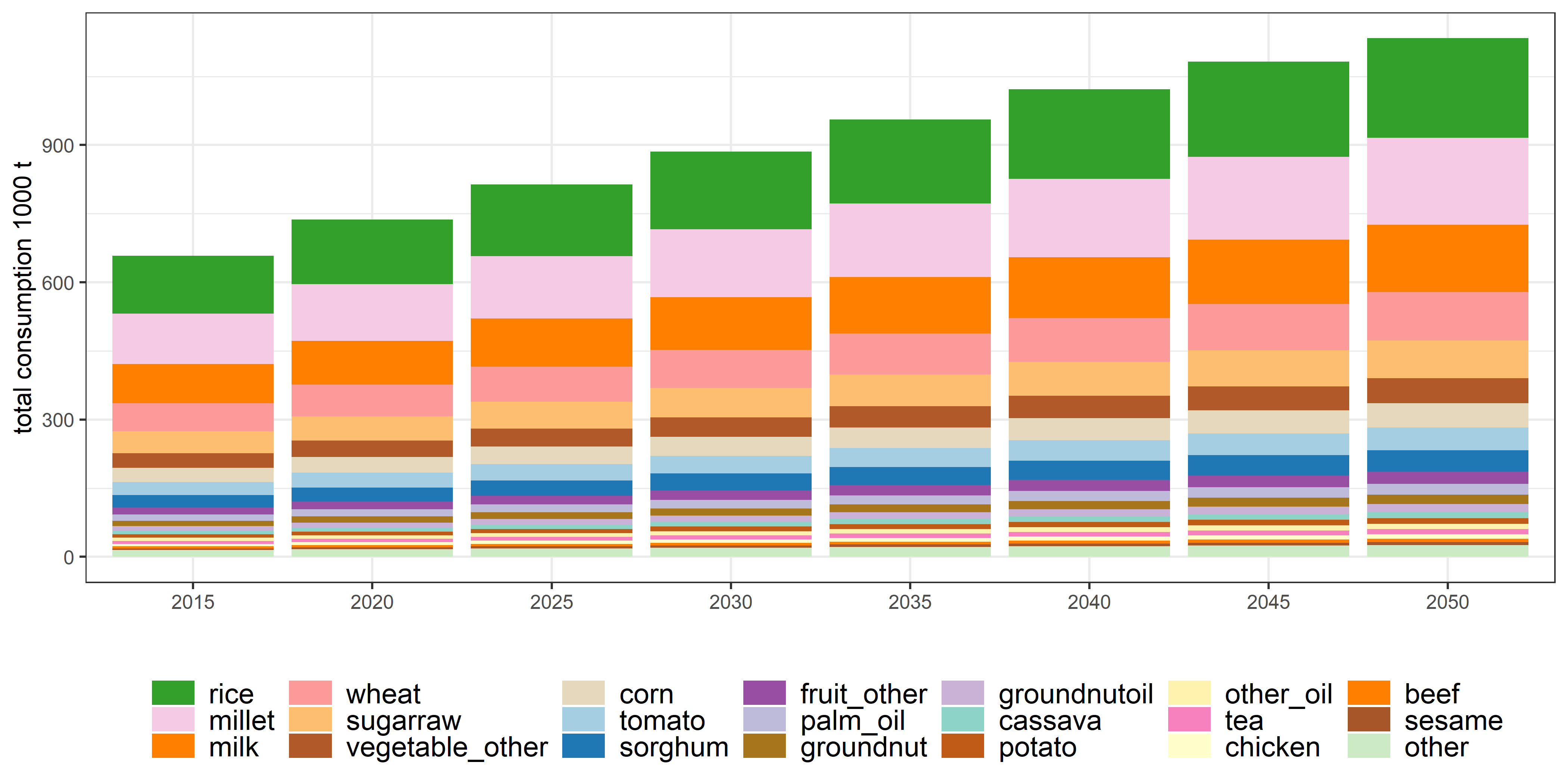


Figure S 5: Estimated change in food demand from 2015 to 2050 under the baseline scenario for the 20 most consumed food groups.


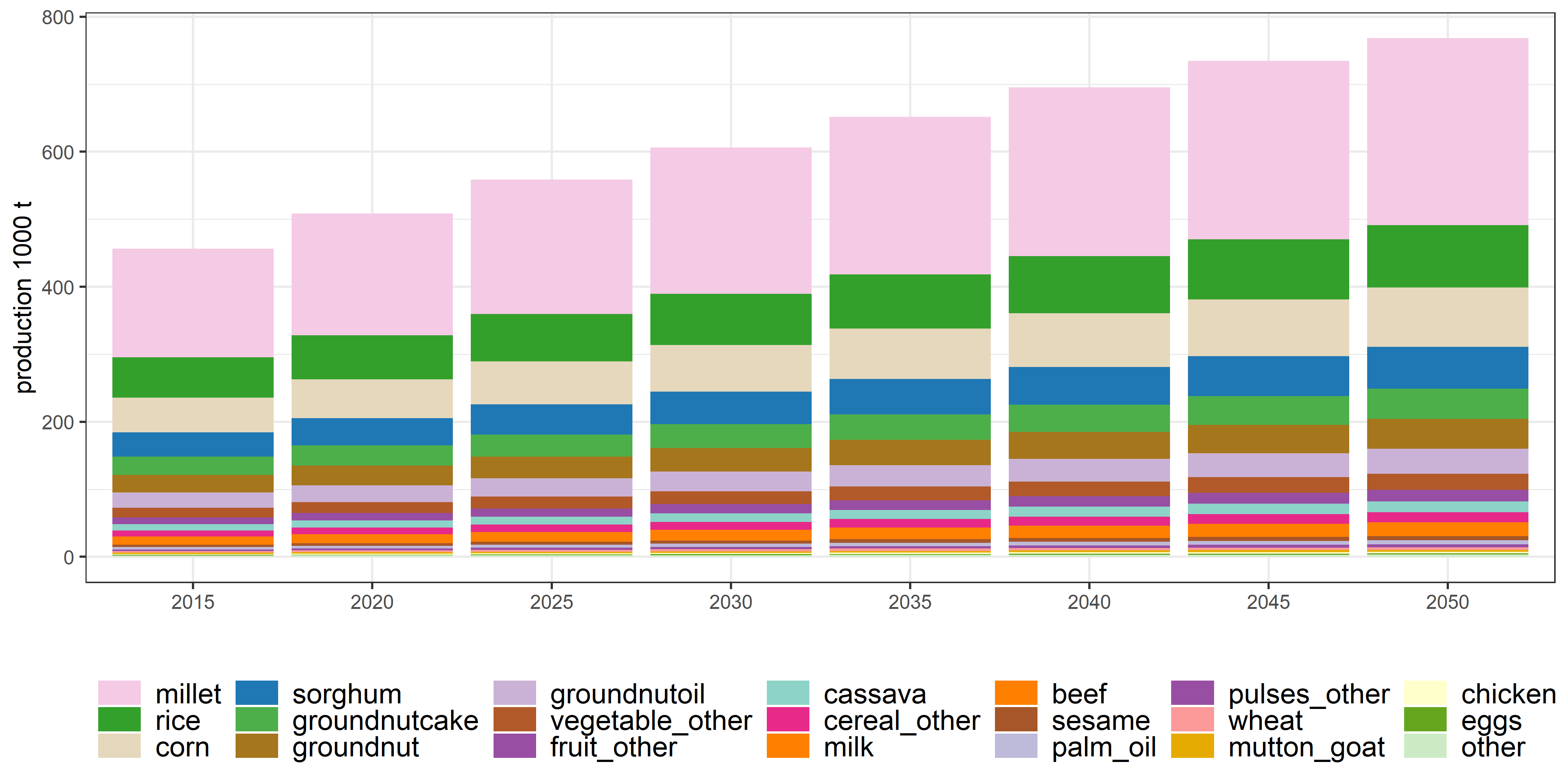


Figure S 6: Estimated domestic food production needed to meet food demand from 2015 to 2050 under the baseline scenario for the 20 most produced food groups.


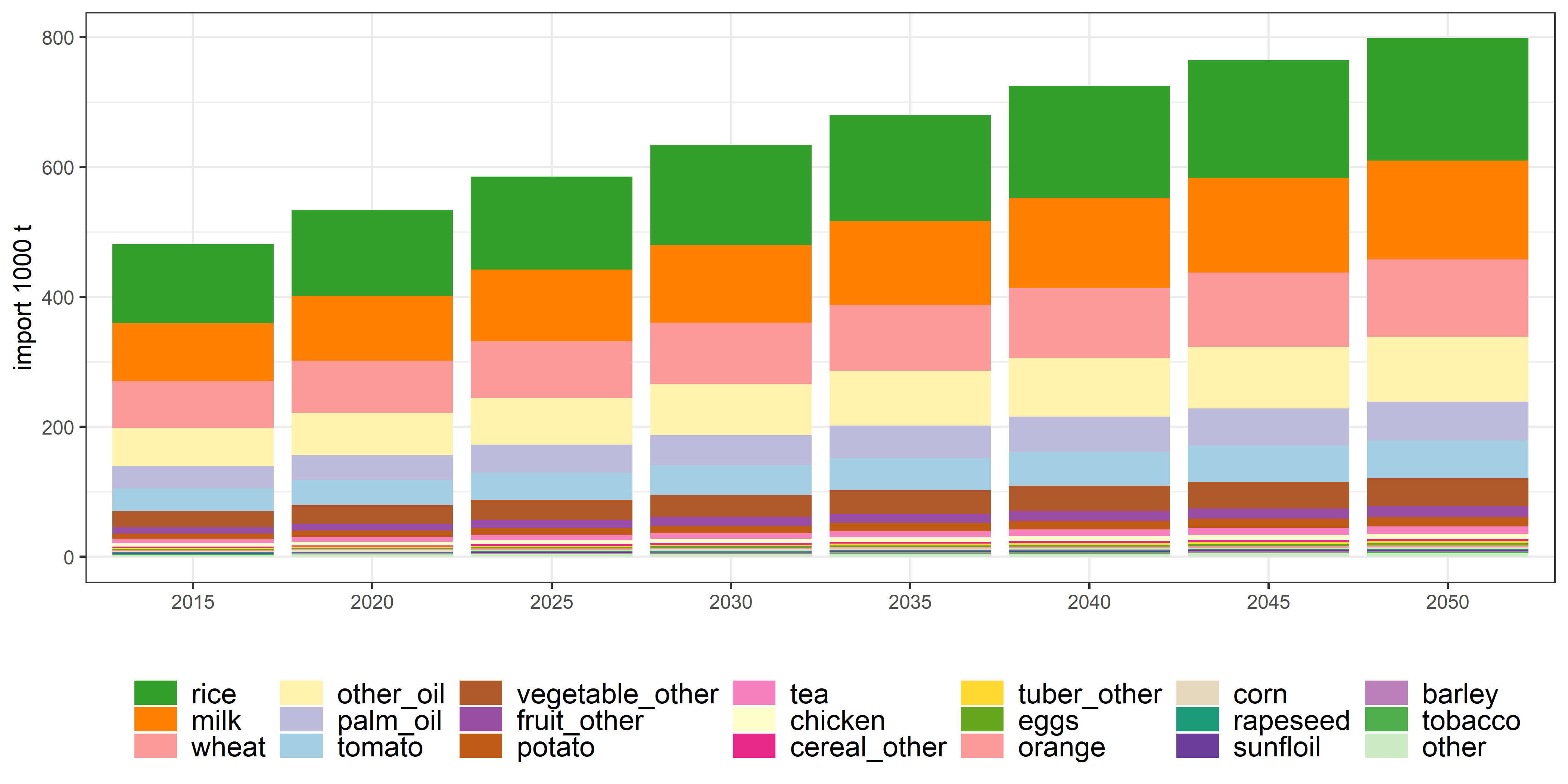


Figure S 7: Estimated food imports needed to meet food demand from 2015 to 2050 under the baseline scenario for the 20 most imported food groups.


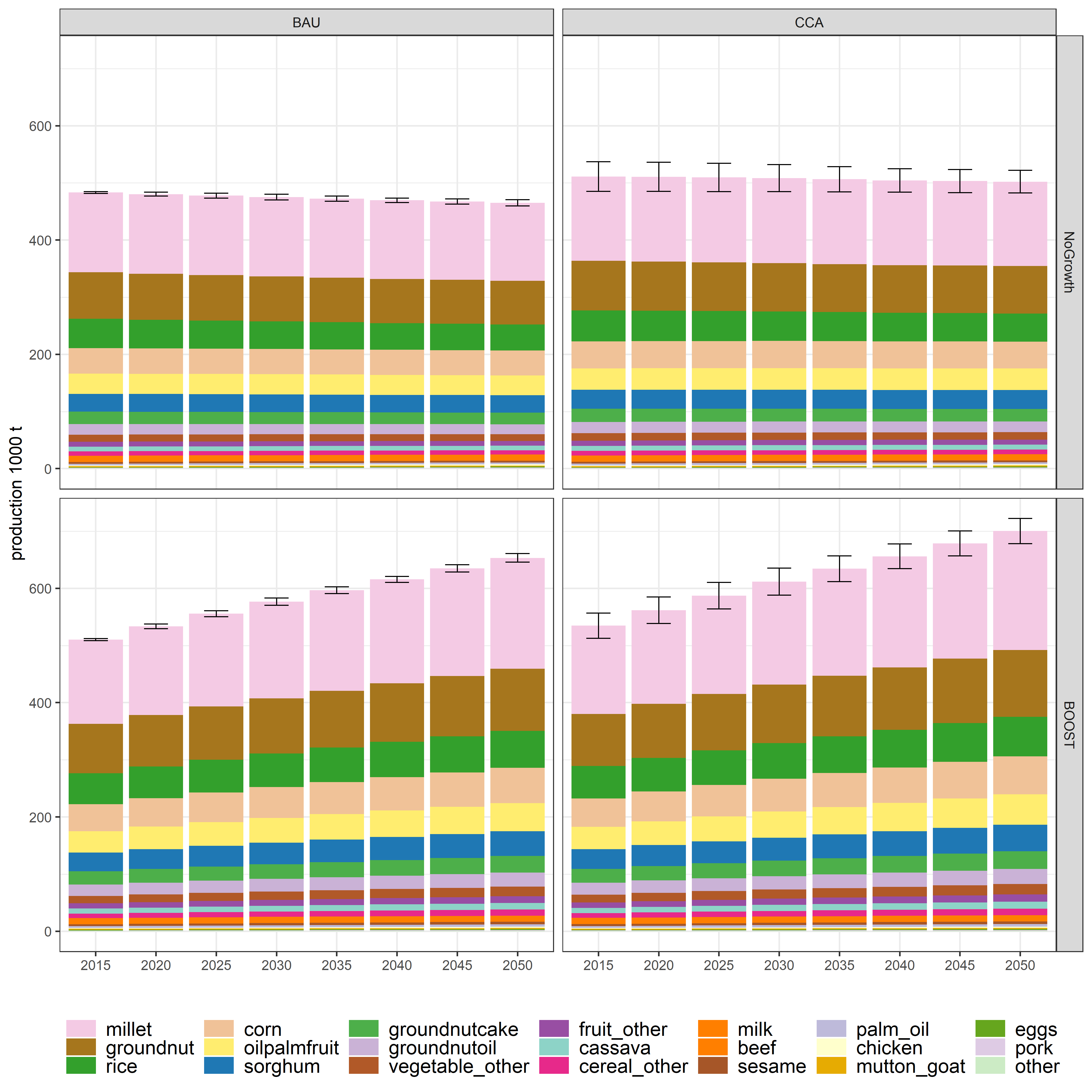


Figure S 8: Estimated feasible domestic food production from 2015 to 2050 for the 20 most produced food groups simulated under different crop productivity scenarios (BAU – business-as-usual, CCA – climate change adaptation, BOOST – intensified nutrient and water management).


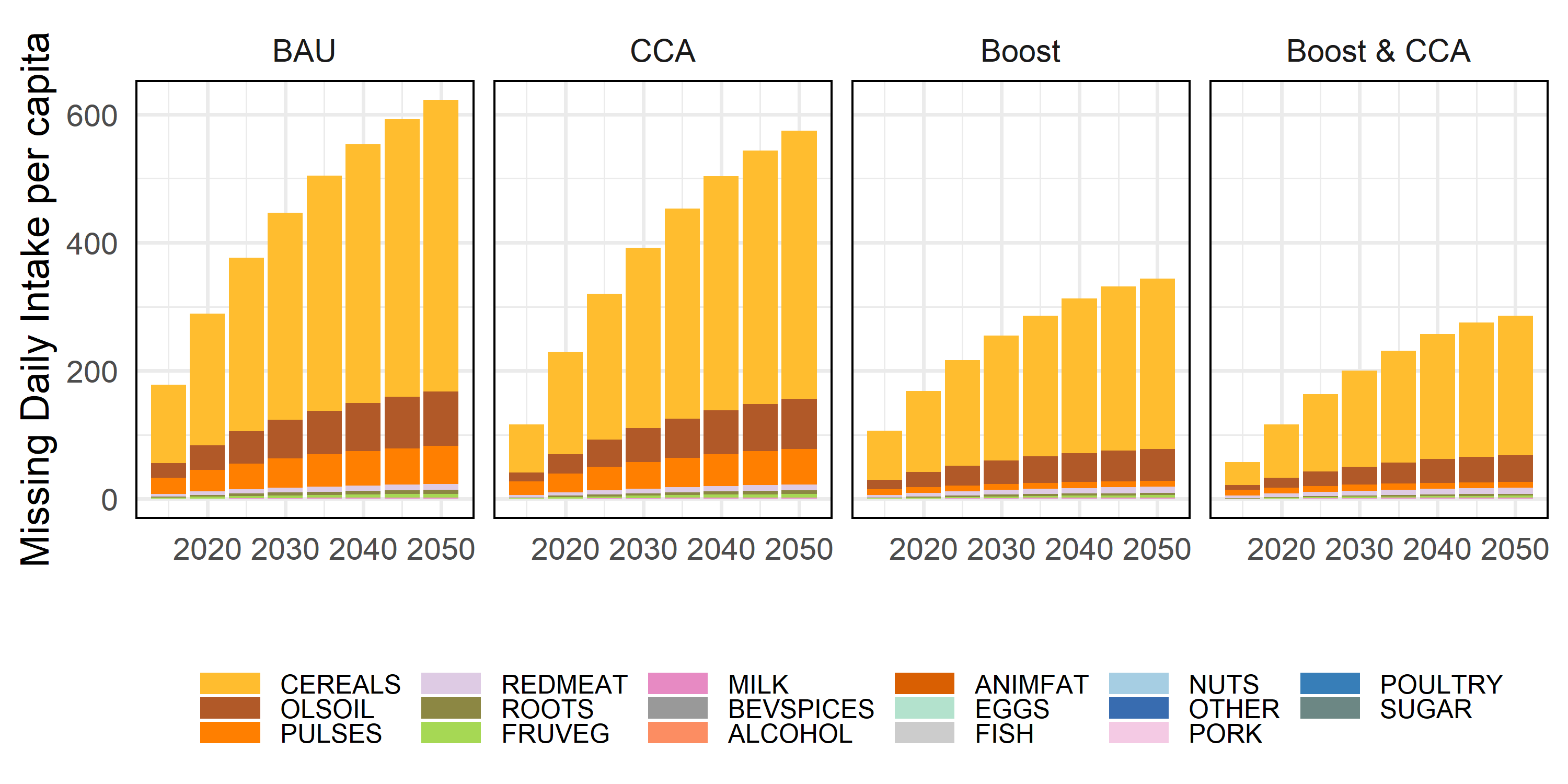


Figure S 9: Missing daily food per capita available for consumption (in kcal) due to the gap between domestic demand and feasible domestic supply from 2015 to 2050 simulated under different crop productivity scenarios (BAU – business-as-usual, CCA – climate change adaptation, BOOST – intensified nutrient and water management).

**References**

Arneth, A., Balkovic, J., Ciais, P., de Wit, A., Deryng, D., Elliott, J., Folberth, C., Glotter, M., Iizumi, T., Izaurralde, R. C., Jones, A. D., Khabarov, N., Lawrence, P., Liu, W., Mitter, H., Müller, C., Olin, S., Pugh, T. A. M., Reddy, A. D., … Büchner, M. (2017). *ISIMIP2a Simulation Data from Agricultural Sector*. GFZ Data Services. https://doi.org/10.5880/PIK.2017.006

Basnet, S., Wood, A., Röös, E., Jansson, T., Fetzer, I., & Gordon, L. (2023). Organic agriculture in a low-emission world: Exploring combined measures to deliver a sustainable food system in Sweden. *Sustainability Science*, *18*(1), 501–519. https://doi.org/10.1007/s11625-022-01279-9

FAO. (2023). *FAOSTAT*. https://www.fao.org/faostat/en/#home

González-Abraham, C., Flores-Santana, C., Rodríguez-Ramírez, S., Olguín-Álvarez, M., Flores-Martínez, A., Torres Rojo, J. M., Bocco Verdinelli, G., Fernández Calleros, C. A., & McCord, G. C. (2023). Long-term pathways analysis to assess the feasibility of sustainable land-use and food systems in Mexico. *Sustainability Science*, *18*(1), 469–484. https://doi.org/10.1007/s11625-022-01243-7

Jha, C. K., Ghosh, R. K., Saxena, S., Singh, V., Mosnier, A., Guzman, K. P., Stevanović, M., Popp, A., & Lotze-Campen, H. (2023). Pathway to achieve a sustainable food and land-use transition in India. *Sustainability Science*, *18*(1), 457–468. https://doi.org/10.1007/s11625-022-01193-0

KC, S., & Lutz, W. (2017). The human core of the shared socioeconomic pathways: Population scenarios by age, sex and level of education for all countries to 2100. *Global Environmental Change*, *42*, 181–192. https://doi.org/10.1016/j.gloenvcha.2014.06.004

Mosnier, A., Penescu, L., Pérez-Guzmán, K., Steinhauser, J., Thomson, M., Douzal, C., & Poncet, J. (2020). *FABLE Calculator 2020 update*. https://doi.org/10.22022/ESM/12-2020.16934

Mueller, N. D., Gerber, J. S., Johnston, M., Ray, D. K., Ramankutty, N., & Foley, J. A. (2012). Closing yield gaps through nutrient and water management. *Nature*, *490*(7419), 254–257. https://doi.org/10.1038/nature11420

Perez-Guzman, K., Imanirareba, D., Jones, S. K., Neubauer, R., Niyitanga, F., & Naramabuye, F. X. (2023). Sustainability implications of Rwanda’s Vision 2050 long-term development strategy. *Sustainability Science*, *18*(1), 485–499. https://doi.org/10.1007/s11625-022-01266-0

Rasche, L., Schneider, U. A., & Steinhauser, J. (2023). A stakeholders’ pathway towards a future land use and food system in Germany. *Sustainability Science*, *18*(1), 441–455. https://doi.org/10.1007/s11625-022-01212-0

Smith, A. C., Harrison, P. A., Leach, N. J., Godfray, H. C. J., Hall, J. W., Jones, S. M., Gall, S. S., & Obersteiner, M. (2023). Sustainable pathways towards climate and biodiversity goals in the UK: The importance of managing land-use synergies and trade-offs. *Sustainability Science*, *18*(1), 521–538. https://doi.org/10.1007/s11625-022-01242-8

Wu, G. C., Baker, J. S., Wade, C. M., McCord, G. C., Fargione, J. E., & Havlik, P. (2023). Contributions of healthier diets and agricultural productivity toward sustainability and climate goals in the United States. *Sustainability Science*, *18*(1), 539–556. https://doi.org/10.1007/s11625-022-01232-w

Zerriffi, H., Reyes, R., & Maloney, A. (2023). Pathways to sustainable land use and food systems in Canada. *Sustainability Science*, *18*(1), 389–406. https://doi.org/10.1007/s11625-022-01213-z

1. An Open-Source version of the FABLE Calculator can be downloaded for free at <https://www.abstract-landscapes.com/fable-calculator> [↑](#footnote-ref-1)
2. https://fableconsortium.org/ [↑](#footnote-ref-2)
3. These parameters influence either the supply- or demand-side of the food and land-use system. Mosnier et al. (2020) present the detailed documentation of the FABLE Calculator. [↑](#footnote-ref-3)
4. The biofuel demand is influenced by the biofuel demand assumptions and is not influenced by population. [↑](#footnote-ref-4)
